# Good Roads

**Published:** 1895-06

**Authors:** 


The Journal's attention has been called to the question of good roads, and its influence sought in behalf of this all-important question throughout this country. It should hardly have been necessary for our attention and support to have been asked for in behalf of this movement, when the success and extent of our work is linked so closely with the aims and purposes of the efforts now being put forth to obtain these necessary adjuncts to every successful and progressive community. Every horse-owner, breeder, and driver is interested; hence every veterinarian, whose income largely depends upon the worth and number of the live-stock in his district, has a deep and yet somewhat selfish interest in the question of good roads. Good roads in a community mean better horses and more of them, and more driving of all kinds; they lead to increased business in all directions. Good roads encourage coaching in all its various forms, and the lessened number of those painful accidents incidental to bad roads means that fewer abandon driving each year, and the better roads attract thousands yearly to this pleasant diversion. Yes, we are heartily in favor of the movement, and the columns of this Journal will be ever ready to second in any and all States the good work so well begun in Massachusetts, and which is now being thoroughly agitated in New York, Pennsylvania, and New Jersey. Let every veterinarian be an agitator as well as supporter of every movement in this direction in his community, and he will aid himself while aiding others, and the intelligence, well-being, and thrift of his locality can be well measured by the extent and keeping of good roads.

